# Exploration of Human Activity Recognition Using a Single Sensor for Stroke Survivors and Able-Bodied People

**DOI:** 10.3390/s21030799

**Published:** 2021-01-26

**Authors:** Long Meng, Anjing Zhang, Chen Chen, Xingwei Wang, Xinyu Jiang, Linkai Tao, Jiahao Fan, Xuejiao Wu, Chenyun Dai, Yiyuan Zhang, Bart Vanrumste, Toshiyo Tamura, Wei Chen

**Affiliations:** 1 Department of Electronic Engineering, School of Information Science and Technology, Fudan University, Shanghai 200438, China; lmeng18@fudan.edu.cn (L.M.); 19210720148@fudan.edu.cn (X.W.); jiangxy18@fudan.edu.cn (X.J.); tlkripple@gmail.com (L.T.); 18110720059@fudan.edu.cn (J.F.); chenyundai@fudan.edu.cn (C.D.); 2 Department of Neurological Rehabilitation Medicine, The First Rehabilitation Hospital of Shanghai, Kongjiang Branch, Shanghai 200093, China; 3 Human Phenome Institute, Fudan University, Shanghai 201203, China; 4 Department of Industrial Design, Eindhoven University of Technology, PO Box 513, 5600 MB Eindhoven, AZ, The Netherlands; 5 Center of Rehabilitation Therapy, The First Rehabilitation Hospital of Shanghai, Shanghai 200090, China; wxj11j@163.com; 6 e-Media Research Lab, Campus Group T, KU Leuven, 3000 Leuven, Belgium; yiyuan.zhang@kuleuven.be (Y.Z.); bart.vanrumste@kuleuven.be (B.V.); 7 ESAT-STADIUS, Department of Electrical Engineering, KU Leuven, 3000 Leuven, Belgium; 8 Future Robotics Organization, Waseda University, 1-104, Totsuka-tyou, Shinjuku-ku, Tokyo 169-8050, Japan; tamurat@aoni.waseda.jp

**Keywords:** daily activity recognition, single wearable sensor, stroke, sensor placement

## Abstract

Commonly used sensors like accelerometers, gyroscopes, surface electromyography sensors, etc., which provide a convenient and practical solution for human activity recognition (HAR), have gained extensive attention. However, which kind of sensor can provide adequate information in achieving a satisfactory performance, or whether the position of a single sensor would play a significant effect on the performance in HAR are sparsely studied. In this paper, a comparative study to fully investigate the performance of the aforementioned sensors for classifying four activities (walking, tooth brushing, face washing, drinking) is explored. Sensors are spatially distributed over the human body, and subjects are categorized into three groups (able-bodied people, stroke survivors, and the union of both). Performances of using accelerometer, gyroscope, sEMG, and their combination in each group are evaluated by adopting the Support Vector Machine classifier with the Leave-One-Subject-Out Cross-Validation technique, and the optimal sensor position for each kind of sensor is presented based on the accuracy. Experimental results show that using the accelerometer could obtain the best performance in each group. The highest accuracy of HAR involving stroke survivors was 95.84 ± 1.75% (mean ± standard error), achieved by the accelerometer attached to the extensor carpi ulnaris. Furthermore, taking the practical application of HAR into consideration, a novel approach to distinguish various activities of stroke survivors based on a pre-trained HAR model built on healthy subjects is proposed, the highest accuracy of which is 77.89 ± 4.81% (mean ± standard error) with the accelerometer attached to the extensor carpi ulnaris.

## 1. Introduction

There are 15 million people suffering from stroke globally each year [1]. One of the most post-stroke relevant symptoms is hemiparesis, causing abnormality in movements of one side of the body, which greatly affects the activities of daily living (ADLs) of post-stroke survivors [2,3]. Using impaired limbs to perform ADLs helps the rehabilitation of stroke survivors outside of the hospital [3]. However, stroke survivors are asked to report ADLs by specific questionnaires, which is quite subjective [3,4]. Therefore, an automatic and non-invasive recording approach for human activity recognition (HAR) is needed.

With the fast development of Internet of Things technologies, recent years have witnessed the great advance of HAR within a wide application range. The significant progress in micro-electromechanical systems, wireless communications, and battery technologies has paved the way for the smart, convenient, and cost-effective approach for HAR application [5,6,7]. Basically, there are three kinds of HAR data collection methods: wearable sensor-based method, video-based method, and ambient sensor-based method [8]. Although unobtrusive monitoring can be fulfilled by a video-based method, one prominent concern is the privacy disclosure of users. Furthermore, the shooting range of a camera is limited, more cameras are costly but needed to track the user activities [9], and it is impossible for the HAR in a privacy space, such as the bathroom. By contrast, the ambient sensor-based method embeds varieties of sensors (such as pressure sensors, object sensors, door sensors, etc.) into a domestic environment. Accordingly, various activity-related information is collected, for example, interactions with sensor-embedded objects (the chair embedded with a pressure sensor to detect whether a person is sitting on it, etc.), movements performed by the user [8]. A higher level of privacy can be ensured compared with the video-based method. However, HAR only works in a restricted area [10] and is limited to the sensor attached objects [11]. The wearable sensor-based method can settle the aforementioned shortcomings by adopting wireless sensors in an unobtrusive way [12].

As to the wearable sensor-based method, people wear the sensors on the body, and real-time data can be collected [13]. Wearable sensors, such as surface electromyography (sEMG) sensors, accelerometers (ACC), and gyroscopes (GYRO), have been widely utilized in HAR [14]. The acceleration measured by accelerometers and the angular velocity measured by gyroscopes indicate the motion information. Meanwhile, sEMG records the electrical potential induced by motor units, reflecting the patterns of muscle activation, and can be further utilized to distinguish the passive and active movements [14,15]. Studies showed HAR accuracy could be improved by adopting multiple sensors [16,17]. Nevertheless, overusing sensors leads to extra cost, inconvenience in sensor configuration, and high dimensional data issues, such as more computational complexity, storage space, and communication channels [18].

Studies focusing on HAR with one single sensor are getting more and more attention. For example, Lu et al. [19] utilized one tri-axial accelerometer on the wrist for classifying six kinds of human activities, achieving an average accuracy of 96%. Tian et al. [20] recognized seven activities with an accelerometer on the waist, reaching a mean accuracy of 94.79%. Mane et al. [21] used a single channel sEMG detector for three kinds of hand activity recognition, yielding an accuracy of 93.25%. However, which kind of sensor can provide adequate information in achieving a satisfactory performance, or whether the position of a single sensor would significantly affect the performance has not yet been studied. Cleland et al. [22] employed 8 healthy male subjects with 6 accelerometers placed on the chest, lower back, left thigh, left wrist, left hip, and left foot to perform 7 activities: walking, sitting, lying, standing, running on a motorized treadmill, and walking up and down stairs. They concluded the sensor placed on the left hip achieving the highest F-measure (0.978) by using the SVM classifier with 10-fold cross-validation. Olguin et al. [23] studied the best location using three accelerometers attached to the right wrist, left hip, and chest by performing 8 activities: walking, running, sitting down, standing, lying down (on the chest), hand movements (while standing), crawling, and squatting. The Hidden Markov Model was adopted to classify the activities with 9-fold cross-validation. The best mean accuracy (65.68%) was achieved by using the accelerometer attached to the left hip. Maurer et al. [24] used a watch consisting of an accelerometer and a light sensor to collect data. Six healthy subjects were recruited to perform walking, running, sitting, standing, ascending, and descending stairs with six watches placed on the bag, wrist, shirt, belt, necklace, pocket for each person. The Decision Tree classifier with 5-fold cross-validation was used to classify the activities. They found the watch on the wrist achieving the highest mean accuracy (89%).

To the best of our knowledge, this is the first comparative study to fully investigate the performance of a single sensor like the accelerometer, gyroscope, and sEMG collector on both healthy subjects and stroke survivors and the optimal sensor placement on the body. Twenty-three subjects (9 stroke survivors and 14 healthy people) were recruited for the experiment. Due to motor impairment, stroke survivors can not fulfill most ADLs with ease. We specially selected four ADLs: walking, tooth brushing, face washing, and drinking as our tasks, which could be potentially used for stroke impairment evaluation based on the four activities without recording the sequence of performing the activities. The data were collected using 12 delsys modules, each of which contained a tri-axial accelerometer, a tri-axial gyroscope, and an sEMG collector. The performance of each sensor and their combination (COMB) were evaluated. To be specific, HAR applications under two scenarios were explored. The first was to categorize the dataset into three kinds, i.e., dataset of stroke survivors, dataset of healthy people, and dataset of all subjects. The second was to distinguish the activities of stroke survivors using a generic model built on healthy subjects. In both scenarios, the optimal sensor placement among 12 modules spatially distributed over the upper and lower limb was presented. We adopted Support Vector Machine (SVM) with Radial Basis Function kernel as the classifier, compared with linear SVM and 7 other different commonly used classifiers. In order to obtain an objective result, we adopted the Leave-One-Subject-Out Cross-Validation (LOSOCV) technique to train and assess classification models. The main contributions of the paper are:

- A comprehensive survey on human activity recognition along with various prevailing sensors, namely, the accelerometer, the gyroscope, and sEMG, was investigated.
- The optimal position of each sensor that can obtain satisfactory performance in HAR was provided.
- The feasibility of a pre-trained HAR model built on healthy people for distinguishing the activities of stroke survivors was verified.

## 2. Materials and Methods

### 2.1. Materials

#### 2.1.1. Subject Information

Brunnstrom stage (BS) is a widely used standard for the assessment of stroke-induced motor impairment [25]. Six levels of BS from I (the most serious level) to VI (the lightest level) can represent the impairment severity of the upper and lower extremity. Stroke survivors at BS I and BS II are not qualified to move their limbs voluntarily. As to subjects at BS III, they start to regain the ability to move their limbs voluntarily, but with a very limited range of motions [26]. Furthermore, the high muscle spasticity hinders them from relaxing the muscle and completing the movements [26]. Therefore, stroke survivors at BS III or lower were excluded in our experiment. For stroke survivors at BS IV or above, although the synergy pattern still exists, due to the significant increment of their muscle strength and endurance in most cases, a better muscle control enables them to finish the movements at a higher standard with some consistency [26]. Stroke survivors at BS VI are very rare in hospitals, and they can perform ADLs almost the same as healthy people [27]. To some extent, the participation of healthy subjects acts as that of stroke survivors at BS VI. Therefore, stroke survivors at BS VI were excluded from the group of stroke survivors in this study. For each stroke survivor we recruited, the BS levels of the upper and the lower limb were the same. Totally, we recruited 23 subjects (10 females and 13 males; aged at 42 ± 20.94 years old), including 7 subjects at BS V, 2 at BS IV, and 14 healthy subjects. All healthy subjects were right-handed, which is consistent with the side they used to perform the experiment. While eight stroke survivors were right-handed and one at BS V was left-handed. Both two stroke survivors at BS IV performed the experiment with their right affected side, which was the same for two stroke survivors at BS V (including the left-handed one). Others at BS V participated in the experiment with their left affected side. As to the time they participated in the experiment, six subjects were in the morning, seven in the afternoon, and ten at night. Each volunteer signed the informed consents before participation. The selection criteria for stroke survivors include:

- Abilities to complete ADLs involved in this experiment independently without any assistant.
- No perceptual, cognitive, or communication problem.
- No major post-stroke complication.

The selection criterion for able-bodied subjects was that they did not have any musculoskeletal-related or neurological diseases before.

#### 2.1.2. Data Collection

**Figure 1:**
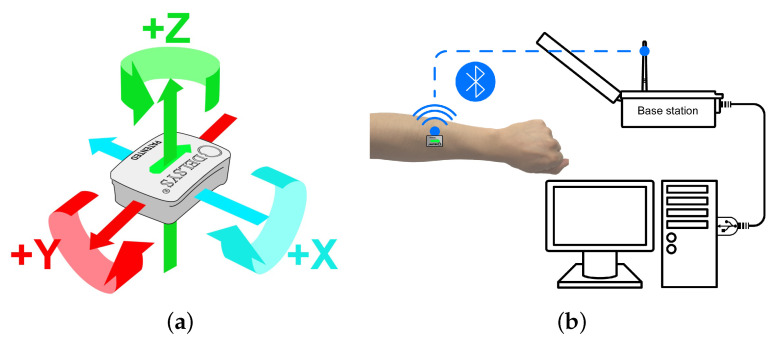
Delsys system. (**a**) Inertial sensing Axis. (**b**) Process of data transmission.

The research was approved by the Ethics Committee of Shanghai First Rehabilitation Hospital, Shanghai, China (HEC No. YK20190409-1-005). The movement signals of ADLs were collected by Delsys wireless trigno system ( Delsys Inc., Boston, MA, USA) containing a base and wireless modules. The delsys system is one of the most commonly used commercial sensor in the world, and each module contains three different kinds of sensors (accelerometer, gyroscope, and sEMG detector). Therefore, three different kinds of data (sEMG, acceleration, and angular velocity) could be collected simultaneously. Information from the three different kinds of data can interpret an activity from different aspects and provide comprehensive information for analyzing the activities, the classification performance of which could act as a benchmark to compare the that of using a single sensor. For the purpose of investigating the optimal sensor and related position for classifying four activities, the same module with different sensors is a compact configuration and approach which uses one module to measure multiple modalities of the activity data. Figure 1a shows the axis of inertial sensors (accelerometer, gyroscope), Figure 1b shows data transmission through one module, the base, and the computer.

**Figure 2:**
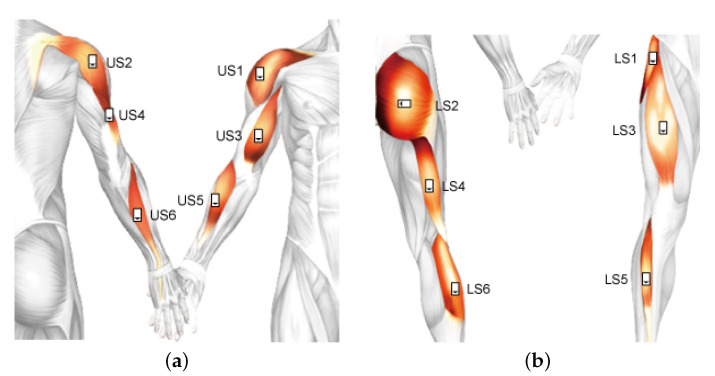
Module positions on the body. (**a**) Module positions on the right upper limb. (**b**) Module positions on the right lower limb. The module direction from the white blank to the black triangle was consistent with the green arrow direction on the module surface of Figure 1a. The actual module placement was placed on the dominant side of healthy subjects and the affected side stroke survivors.

Considering the main body parts used in ADLs, we specially selected 12 key positions of the body to place delsys modules. The label abbreviation of each module and corresponding adherent muscle were illustrated in Table 1. As stroke survivors need to practice their paralyzed limbs and the information of impaired could be used for further exploration of stroke motor function evaluation, modules were placed on the dominant side of healthy subjects and the affected side of stroke survivors after skin preparation (light skin abrasion with Nuprep Skin Prep Gel, cleansing with 70% ethanol). In real life, a feasible solution for placing modules is to follow a picture (shown in Figure 2) with modules in specific positions. The sampling frequency of accelerometers and gyroscopes were set to 148 Hz. Meanwhile, the sampling frequency of sEMG was set to 1260 Hz.

**Figure 3:**
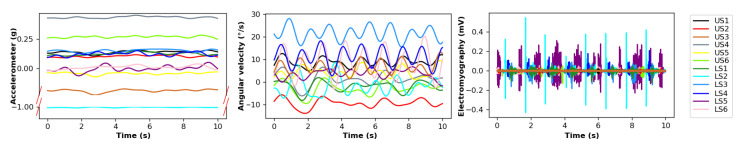
Raw signals of each sensor position using the x-axis of accelerometers, x-axis of gyroscopes, and surface electromyography (sEMG). The space between red slashes on the y-axis were ignored.

**Figure 4:**
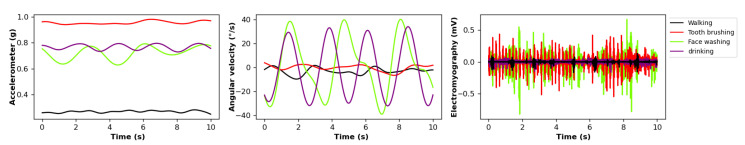
Raw signals of four activities using the x-axis of accelerometer, x-axis of gyroscope, and sEMG of US6.

In order to intuitively observe the data differences among the 12 sensor positions, set the walking as the example, we draw the raw data picture of each sensor position using the x-axis of accelerometers, x-axis of gyroscopes, and sEMG (shown in Figure 3). Furthermore, the raw signals of four activities using the x-axis of accelerometer, x-axis of gyroscope, and sEMG of US6 were presented in Figure 4.

**Figure 5:**
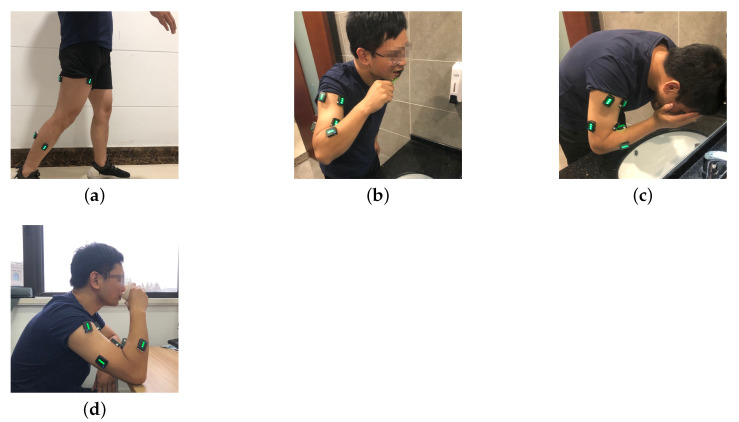
(**a**) Walking. (**b**) Tooth brushing. (**c**) Face washing. (**d**) Drinking.

Considering the daily movements related to the upper or lower extremity were more difficult for stroke survivors using the hemiplegia side than the able-bodied subject due to the motor impairment induced by stroke, we specially selected four ADLs necessary for life as our tasks (tooth brushing, face washing, drinking, and walking) involving movements of the upper and lower limb. The pictures taken in the experiment for one subject were shown in Figure 5. The automatic classification of the four activities can be further used to evaluate the stroke impairment without having to record the sequence of performing the activities. Healthy subjects were required to use the dominant side, while stroke survivors were asked to use the affected side to perform the four kinds of ADLs. There were three trials for each activity, and each trial lasted for 1 min. A one-minute interval was set between trials. Subjects were allowed to rest whenever they felt fatigued, and they could stop the experiment they were doing if they felt unable or in no mood to continue.

Requirements for doing the designated ADLs are as follows:

- Walking: subjects walked straight at their normal walking speed without any assisted tools, such as the stick.
- Tooth brushing: subjects kept brushing their teeth without a break.
- Face washing: two steps were taken to fulfill a round of washing face. The first step was to clean the towel, lasting about 2 s. The second step was to wash the face, lasting about 3 s. The two steps were repeated until the end of the scheduled time.
- Drinking: Subjects picked up the cup to drink. The duration of drinking lasted about 2 s. Then, subjects put down the cup on the table and started to pick up the cup to drink 1 s later.

In order to get close to the practical application, we respected subjects’ living habits. The requirements were for reference only, they could adjust the ADLs requirements based on their habits. For example, they were free to use the towel or nor for washing face and allocate the duration of each step within each trial of face washing and drinking.

### 2.2. Methods

**Figure 6:**
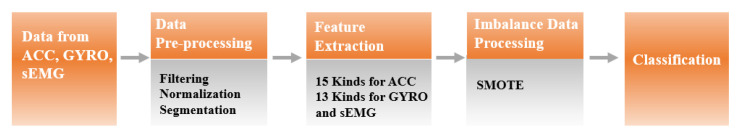
The block diagram of the assessment framework.

The overall system flowchart was shown in Figure 6. First, the collected data were filtered to eliminate the surrounding noise. Then, signals of each activity were normalized by z-score normalization, and different kinds of features were extracted after data preprocessing. Due to the physical or mental difficulty in keeping performing activities for stroke survivors, the number of data segments was less than the set number for several trials, leading to imbalanced data. Therefore, we adopted the Synthetic Minority Over-sampling Technique (SMOTE) on the training set to solve the problem. At last, the optimal sensor and the corresponding placement was determined through the following classification method.

#### 2.2.1. Data Pre-Processing

The data collected from accelerometers and gyroscopes were low-pass filtered with a fourth-order Butterworth filter (cut-off frequency: 5 Hz), while the sEMG signal was band-pass filtered with a fourth-order Butterworth filter (cut-off frequency: 10–500 Hz) to eliminate the surrounding noise [27]. The Z-score normalization method was used for signals of each activity. The fixed-size window technique (the most common data segmentation method [28]) was adopted to segment the data stream into fixed length with no gap between adjacent windows. 10-second window length with no overlap strategy was adopted in this study.

#### 2.2.2. Feature Extraction

Time-domain features have been widely applied in the biomedical engineering field. As no transformation is required in the calculation process, time-domain features are qualified with the advantage of easy and quick implementation [29]. There are three accelerometer channels (x-, y-, z-axes), three gyroscope channels (x-, y-, z-axes), and one sEMG channel for each module. Thirteen common features were extracted for each channel, including mean, median, variance, absolute energy, root mean square, standard deviation, skewness, kurtosis, zero crossing, sample entropy, slope sign changes, interquartile range, mean absolute value [30,31,32,33,34]. Two complementary features (maximum jerk and mean jerk) [34,35] were extracted for accelerometers. Therefore, 45 (3 channels × 15 features) were extracted for one single accelerometer, 39 (3 channels × 13 common features) features for one gyroscope, 13 (1 channel × 13 common features) features for one sEMG sensor, and 97 (45/accelerometer + 39/gyroscope +13/sEMG) features for the combination of one module. The extracted for each sensor and their combination were used for the following analysis.

#### 2.2.3. Imbalanced Data Processing

Subjects suffering stroke could try to control their hemiplegic side of their bodies to perform ADLs like able-bodied people. They tend to feel more fatigued (physically or mentally) than healthy people in the process of doing the same task. Therefore, subjects were allowed to end activities at any time. As there was a three-minute effective data stream for each activity and the segment length was 10 s, the total setting number of segments of each activity was 18 (60 s/10 s × 3 trials × 1 activity). All healthy subjects fulfill the scheduled experiment. However, a couple of stroke survivors ended part of the trials in advance, leading to a class imbalance in the training set. To be specific, all stroke survivors finished the designed walking experiment. One stroke survivor at BS IV ended tooth brushing and face washing in advance, and the number of data segments of the subject’s tooth brushing and face washing were 9 and 11, respectively. Another one stroke survivor at BS V also quit face washing, the collected segment number of which was 16. In addition, two different stroke survivors at BS V ended drinking ahead of schedule, and the number of data segments were 8 and 17, respectively. Chawla et al. [36] proposed a heuristic oversampling technology: SMOTE, which has been widely used in processing class imbalance [37]. To balance the dataset, we adopted SMOTE for better recognition of minority classes. The key step of SMOTE is to linearly interpolate new points randomly between samples of a minority class and their neighbors. Suppose *c* was the minority class we intended to balance, the SMOTE could be explained mathematically as follow:

(1) p=x+w(y−x)

where *x* denotes a point from the minority class *c*; The typical and default value of *K* is 5 [38,39,40], so we adopted 5 nearest neighbors in this study. Based on the distance from *x*, 5 nearest neighbors of *x* from the same class *c* are chosen [38]; *y* is the point randomly selected from the 5 nearest neighbors, *x* and *y* are from the same minority class *c*; *p* denotes a newly interpolated point (class *c*).

We took advantage of SMOTE on the training set to solve the imbalance issue.

#### 2.2.4. Classification

To investigate the performance of using one single sensor (accelerometer, gyroscope, and sEMG sensor) and the optimal position, we comprehensively compare the results under two different scenarios. The first scenario was to divide the dataset into three groups based on the kind of subjects recruited, i.e., stroke survivors, healthy subjects, and all subjects. Accordingly, the effects of different kinds of sensors on the HAR performance among different groups could be comprehensively evaluated based on the mean accuracy. The accuracy for each person was defined as the number of activities correctly classified divided by the number of all activities performed by the person. In each group, we adopted the LOSOCV technique to train and assess classification models. One step of LOSOCV is that one subject is left for testing the trained model, while the rest are used for training the classification model. The step is repeated until each subject has been used for testing the trained model. Instead of biased estimation of performance by other validation methods, LOSOCV offers an objective and realistic result when the trained classification model is tested on a new subject.

The second scenario was to train the model using the dataset of healthy subjects while test the model using the dataset of stroke survivors, which was more meaningful in practical application. The reported mean accuracy, accordingly, was utilized as the evaluation index.

In all cases, we utilized the SVM with the Radial Basis Function (RBF) kernel as the classifier. C and gamma were two important hyperparameters of SVM. We tuned the value of C (1, 10, or 100) and gamma (1, 0.1, or 0.01) for training the model by the grid search method. As a comparison, we selected linear SVM and 7 different commonly used machine learning classifiers with validated parameters in other HAR studies: (1) K-Nearest Neighbor (KNN) [41], (2) Decision Tree (DT) [42], (3) Adaboost [43], (4) Random Forest (RF) [44], (5) Linear Discriminant Analysis (LDA) [45], (6) Artificial Neural Network (ANN) [46], (7) Gaussian Naive Bayes (GNB) [47], and (8) Linear SVM [48]. To be specific, the C4.5 algorithm was selected for DT, 5-nearest neighbors were set for KNN, the number of Decision Tree classifiers in Random Forests was 30, the maximum number of estimators for Adaboost was 50. ANN with the structure of (50, 1) was investigated. As for the feature selection, The minimum-redundancy maximum-relevancy (mRMR) [49] was adopted as the feature selection method to prune the number of features and improve the accuracy of evaluation systems [50]. mRMR is a kind of mutual-information-based feature selection, which combines the criteria of Max-Relevance and Min-Redundancy. Mutual information is an approach to characterize the relevance of two variables. MI has the ability to measure various kinds of relations between variables, including nonlinear relations [51]. As to discrete variables, given two discrete random variables *X* and *Y*, the MI is defined as follows:

(2) $$I\left(X;Y\right)=\sum_{x\in X}\sum_{y\in Y}p\left(x,y\right)log\left(\frac{p(x,y)}{p(x)p(y)}\right).$$

where p(x), p(y), and p(x,y) denote probabilistic density functions of *x*, *y*, the joint probability of *x* and *y*, respectively.

The aim of Max-Relevance is to choose features having the highest relevance to the target class *c*, which could be fulfilled by searching a feature subset *S* satisfying (3) mathematically. To be specific, suppose we have *m* features in total. The first ranked feature is the one maximizing I(xi;c)(i=1,2,...,m). Then, the forward stepwise search scheme is adopted to add another one feature from the remaining features. The one maximizing *D* in Formula (3) is ranked as the second. The step is repeated until no feature is left.

(3) $$max\;D\left(S,c\right),\;D\left(S,c\right)=\frac{1}{\left|S\right|}\sum_{f_{i}\in S}I\left(f_{i};c\right).$$

where $f_{i}$ denotes an individual feature, *c* denotes a class.

However, using the combinations of individually good features can not always ensure a good classification accuracy. As a result of the dependency among features, the feature subset selected by the Max-Relevance could have rich redundancy, leading to a degradation of classification performance. Therefore, the Min-Redundancy criterion is introduced to deal with the redundancy problem by excluding dependent features [52]:

(4) $$min\;R\left(S\right),\;R\left(S\right)=\frac{1}{{\left|S\right|}^{2}}\sum_{f_{i},f_{j}}^{}I\left(f_{i},f_{j}\right).$$

The criteria of combining the Max-Relevance and Min-Redundancy constraints is called mRMR. The operator F(D,R) is defined to combine *D* and *R*, which are optimized simultaneously following the Formula (5):

(5) maxΦ(D,R),Φ=D−R

To evaluate the performance of a certain sensor, we selected the highest accuracy among the accuracies obtained using the top *n*(*n* = 1, 2, …, the number of all features) features as the best performance of the certain sensor.

#### 2.2.5. Statistics Analysis

The main objective of this study was to investigate the performance of one single sensor from different kinds. To explore whether different sensors and their combination had any effect on the final results under the first scenario, the one-way Friedman test was conducted on the accuracies. The factor was the kind of single sensor and the combination of the three kinds of sensors. If the overall significant difference was detected, post hoc analysis was applied for pair-wise comparison. The significance level was set to 0.05 for the statistics part.

## 3. Results

To comprehensively evaluate the performance of frequently used sensors for HAR, we studied the HAR performance using the data collected from the acceleration, angular velocity, sEMG, and their combination separately under two scenarios.

### 3.1. Comparison of Different Classifiers

HAR performance using different kinds of classifiers under the two scenarios was presented in Table 2. The SVM classifier with RBF kernel outperformed the rest classifiers except for two scenes. The first scene was using the accelerometer within the group of healthy subjects, the highest classification accuracy of which (96.73%) was achieved by the Adaboost classifier. While the second scene was using the sensor combination within the group of healthy subjects, the highest classification accuracy of which (97.82%) was achieved by the linear SVM classifier. Statistics analysis showed there was no significant difference between the SVM classifier with RBF kernel and Adaboost in the first scene, and between the SVM classifier with RBF kernel and linear SVM in the second scene. Therefore, the following analyses were conducted using the SVM classifier with RBF kernel.

### 3.2. Analyses of the First Scenario

**Figure 7:**
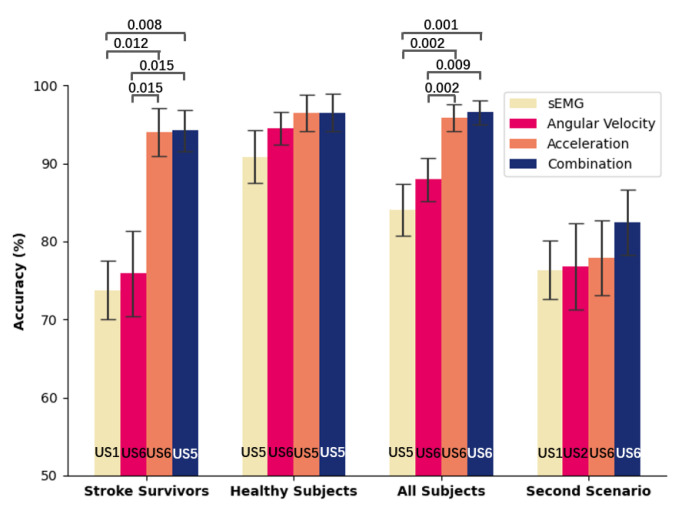
Best human activity recognition (HAR) accuracies (mean ± standard error) using the accelerometer, gyroscope, sEMG collector, and the combination of three kinds of sensors in one delsys module within groups of stroke survivors, group of healthy subjects, and all subjects under the first scenario, and the second scenario. The sensor abbreviation at the bottom of the bar graph denotes the sensor achieving the highest accuracy. The line connection between two sensors indicates there is a significant difference between the two items, and the corresponding *p*-value is shown above the line. No significant difference (*p* > 0.05) is detected between two items if there is no line connection.

Figure 7 showed the best HAR accuracies among the 12 locations for each kind of sensors and their combination, the corresponding sensor utilized, and related statistics. As to the comparison among different subject groups, the performances among the group of healthy subjects were superior to other groups for each kind of sensor, while performances among the group of stroke survivors were at the lowest. Considering the effect of each sensor and the combination on the evaluation performance within each subject group, the highest accuracy was achieved by using the combination. As to one sensor, sEMG achieved the lowest accuracy, while the accelerometer reached the highest accuracy. The highest accuracy for the group of healthy subjects was (96.43 ± 2.35%), obtained by using the accelerometer (US5) attached to the brachioradialis. Meanwhile, using the accelerometer (US6) attached to the extensor carpi ulnaris achieved the highest accuracies for the group of stroke survivors (94.05 ± 3.1%) and the group of all subjects (95.84 ± 1.75%). The performance of using the accelerometer showed no significant difference with that of using the combination for each group.

In each group, statistics analysis of HAR classifications among three different sensors, and the combination showed that there were overall significant differences. To be specific, the *p*-value for the group of stroke survivors and the group of all subjects was less than 0.001. Meanwhile, *p* = 0.014 was for the group of healthy subjects. Then, the post hoc analysis was performed for pair-wise comparisons when an overall significant difference was detected. As a result, no significant difference was observed between items in the group of healthy subjects, while the significant difference distribution between items was the same for the group of stroke survivors and that of all subjects. Specifically, there was no significant difference between the sEMG and the gyroscope, and between the accelerometer and the combination, while the significant difference was discovered between non-aforementioned combinations.

**Figure 8:**
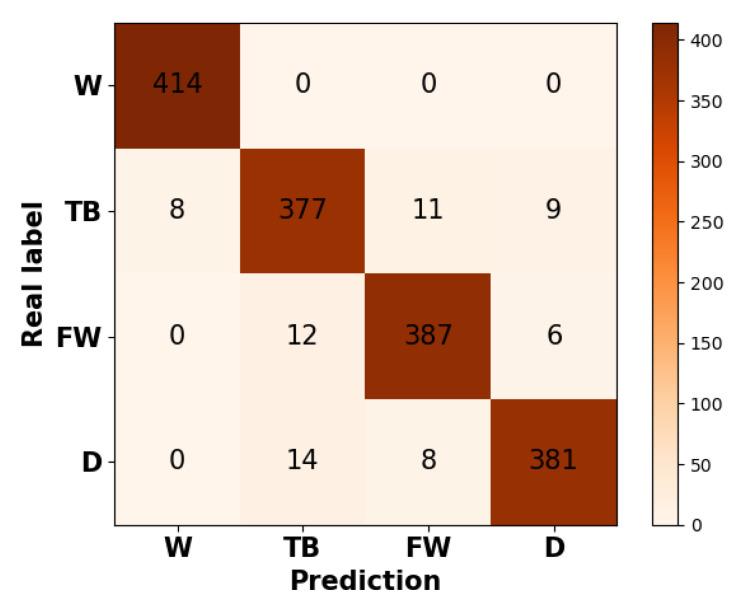
Overall confusion matrix using the accelerometer of US6 for the group of all subjects. W denotes walking; TB denotes tooth brushing; FW denotes face washing; D: denotes drinking.

Figure 8 showed the overall confusion matrix using the accelerometer of US6 for the group of all subjects. We adopted the marco-average method to calculate the overall classification accuracy of each activity. To be more detailed, we first obtained the accuracy of each activity of each individual. Then we averaged the accuracy of each activity. As a result, the classification accuracies of walking, tooth brushing, face washing, and drinking were 100%, 92.75 ± 3.47%, 95.5 ± 1.69%, 94.69 ± 2.58%. In order to explore the HAR performance differences among the subject group of BS IV, BS V, and health, we sorted out the classification accuracy based on the kind of groups. Results showed the mean accuracy was 97.32 ± 2.68% for the group of BS IV, 90.08 ± 4.83% for the group of BS V, and 98.51 ± 1.07% for the group of health.

Considering application involving stroke survivors, we dedicatedly carried out the pair-wise comparison of accelerometers in different sensor placements for the group of all subjects to investigate whether other sensors, except for the best performance sensor (US6), would have a significant effect on the performance. In Table 3, sensors were ranked according to their performances (from the best to the worst) using the acceleration data of all subjects. The accuracy of each sensor was shown in parenthesis below the related sensor in the first row. We could conclude that there was a trend that there was no significant difference between sensors having an adjacent position in Table 3. The accelerometer attached to the brachioradialis (US5) and that attached to rectus femoris (LS3) had no significant difference with US6.

**Figure 9:**
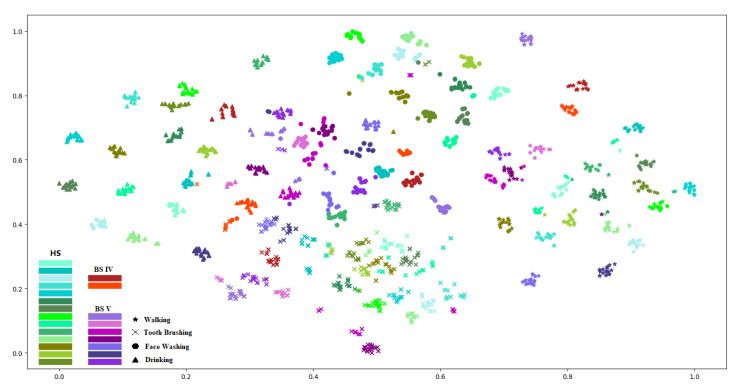
Feature distances for the inter-individual and inter-activity differences.

To further reveal the diverse classification performances, we explored the inter-individual and inter-activity differences, which could be illustrated through the feature differences among individuals and activities. The feature differences could be observed form distance between each feature in a figure intuitively. We utilized t-distributed stochastic neighbor embedding (t-SNE) technology [53] to present the inter-individual and inter-activity feature differences using the features extracted from all 12 modules. The distances between features were presented in Figure 9. We used the extracted features to classify the activities. Accordingly, the classification performance could be affected by the feature differences between each activity. While, the individual feature differences, especially between the stroke survivors and healthy people, could illustrate the accuracy degradation when the stroke survivors were involved in the experiment, and further interpret the different movement patterns between stroke survivors and healthy people.

There was a trend that the features of each individual gathered together, so did the features of each activity. Furthermore, features of stroke survivors in each activity tended to be together, showing there were differences between stroke survivors and healthy subjects.

### 3.3. Analyses of the Second Scenario

We evaluated the performances of using each of the accelerometers, gyroscopes, sEMG collectors, and their combinations under a practical scenario that the model was trained using the dataset of healthy subjects while tested using the data from stroke survivors. Figure 7 shows the highest classification mean accuracy during the 12 sensors for each kind of sensor and the combination, and the optimal sensor placement. Similar to the first scenario, the highest accuracy (82.47 ± 4.18%) was obtained by using the combination, and sEMG achieved the lowest accuracy. The highest accuracy using one sensor was 77.89 ± 4.81% by using the accelerometer (US6) on the extensor carpi ulnaris. Statistics showed there were no overall significant differences (*p* = 0.402) among the three kinds of sensors and their combination.

## 4. Discussion

Although wearing multiple sensors could improve the HAR accuracy, it brings burdens on purchasing sensors, inconvenience in sensor configuration, and high computational complexity data issues. As a result, one-sensor-based HAR is prevailing recently. However, there is no comparative study investigating the performance of various prevailing sensors on HAR involving stroke survivors and the optimal sensor placement on the body. In this study, we investigated HAR performance involving stroke survivors using one single sensor from different positions of the body. The first scenario was to evaluate the HAR performance using the sEMG, accelerometer, gyroscope, and their combination within different subject groups (stroke subjects, healthy subjects, and all subjects). The second scenario was to classify activities of stroke survivors using a pre-trained HAR model built on the dataset of healthy subjects. We aimed to find the best placement and the related single sensor for achieving the same classification performance with the combination of sensors. The accelerometer (US5) placed on the brachioradialis perform the best for the group of healthy subjects in the first scenario, while the accelerometer (US6) placed on the extensor carpi ulnaris can achieve the highest accuracy for the rest situations. For the group of healthy subjects in the first scenario, the accuracy of using the accelerometer (US6) was 95.44 ± 2.71%, and there were no overall significant differences among the performance of using the accelerometer (US5), the accelerometer (US6), and the three sensor combination (US5). Therefore, the accelerometer (US6) attached to the extensor carpi ulnaris is recommended for the HAR. As for a single sensor, experimental results showed that the highest mean accuracy of activity classification (95.84%) involving stroke survivors was achieved by using the accelerometer attached to the extensor carpi. Although the highest mean accuracy of the combination of the accelerometer, gyroscope, and sEMG (96.56%), achieved on the same location (the extensor carpi) was a little higher than that of using the accelerometer (95.84%), there was no significant difference between them. In addition, the sampling rate of the accelerometer is far lower than that of sEMG, which could further lower the power consumption.

As to the sEMG signal, it is dynamic and complicated in nature [54]. In addition, instrument characteristics (such as the electrode deviation), anatomical properties, and physiological factors also affect the sEMG signal, leading to the variation of sEMG property from person to person [54,55]. Such individually unique properties of sEMG characteristics could even be used for user authentication [56]. Meanwhile, the physical disability (caused by the injured brain) of stroke survivors is characterized by loss of strength and dexterity to the afflicted side of the body [57,58]. The signal from the injured brain of stroke survivors for controlling limbs would be abnormal, leading to a larger sEMG variation among different stroke survivors. Therefore, HAR performance using the sEMG sensor was at the lowest. The data collected from the accelerometer and the gyroscope could be used to represent the kinematic information. Compared with sEMG, the kinematic information is only related to the properties of observed motions, which could represent a specific motion pattern, regardless of physiological factors. Therefore, the best HAR performance was achieved by using the accelerometer. The movement patterns of stroke survivors and health subjects are different, resulting in an unsatisfactory accuracy when we apply the pre-trained model built on the healthy subjects to test the stroke survivors. Accordingly, the classification performance would improve when the data of stroke survivors were involved in the training process, resulting in the high HAR accuracy of the group of all subjects under the first scenario. The classification accuracy using the accelerometer of US6 in the group of all subjects for the three healthy levels was: healthy > BS IV > BS V. The potential reason for the higher accuracy of BS IV than BS V was that there were only two stroke survivors at BS IV, which would bring out some accuracy deviation. However, due to the limited number of stroke survivors and the heavy body burden for them to perform ADLs, the data of stroke survivors are very scarce in practice. Meanwhile, we do not need to worry about the number of healthy people, the data can even be collected in a household scene, and we can easily acquire data from healthy subjects on a large scale. In addition to the explanation of the data scarcity of stroke survivors, we could see features of each activity tended to gather together in Figure 9, which indicated there was a possibility to classify the ALDs of stroke survivors with the model built on healthy subjects. We explored the second scenario with the hope of finding the actual classification performance. The practical use value can be greatly improved if the pre-trained model built on the healthy subjects could be used to test the stroke survivors.

To better illustrate the superiority of the HAR performances using one single sensor in our study, we compared the existing works involving stroke survivors. Zhang et al. [59] developed a shoe embedded with a tri-axis accelerometer and force-sensitive resistors for classifying three activities (standing, sitting, and walking), and mean classification accuracy of 91.5% was achieved for the group of 12 stroke survivors. Laudanski et al. [60] used two accelerometers and a gyroscope attached to both sides of shanks averagely for recognizing five kinds of activities (walking, stair ascent, and descent in the step-over-step and step-by-step manners), achieving a mean HAR accuracy of 94% for the group of 10 stroke survivors. Lonini et al. [61] recruited eleven able-bodied subjects and ten stroke survivors to perform five activities (walking, standing, sitting, climbing stairs up and down) with a tri-axial accelerometer on the waist. They achieved a mean HAR accuracy of 54.86% for the group of stroke survivors and 50.32% when a pre-trained model built on healthy subjects was used to test the HAR performance of stroke survivors, which was far from practical uses. Overall, our solution reached the competitive mean classification accuracy of 94.05% for the group of stroke survivors and 77.89% for the HAR classification of stroke survivors using the HAR model training on the dataset of healthy subjects. The mean accuracy could be improved to 95.84% when healthy subjects participated.

This was a preliminary study on exploring the suitable sensor among the accelerometer, gyroscope, and sEMG detector for HAR. Meanwhile, the optimal sensor position on the body was presented. In order to validate whether the reduced number of sensors would sacrifice the accuracy in distinguishing human activities, we compared the results obtained by using a single sensor and the results gained by using all the sensors in the same position. At last, a feasible approach to identify the activities of stroke survivors was proposed. However, this work can still be enhanced by addressing the following issues. First, only four ADLs were involved in the experiments. Second, the stroke survivors we recruited were mainly distributed at BS IV and V due to the limited number and the willingness of hospitalized patients. The presented work has demonstrated promising results of classifying ADLs for both stroke survivors and healthy people. However, extended works can be conducted in the future: (1) More stroke survivors dispersed throughout the BS level should be included to rigorously assess the generalization ability and the acceptability of the single sensor in HAR. (2) We can design HAR experiments involving more activities (such as lying, standing, ascending and descending stairs, etc.) with the more detailed consideration of physical conditions of the stroke survivors at different BS levels to further verify the reliability and the robustness of the proposed method. (3) As the data of stroke survivors are very scarce in practice and the classification will improve using the data combination of healthy people and stroke survivors, we can explore the performance improvement using the data combination of more healthy people and fewer stroke survivors to reach a satisfactory result. (4) In light of the presented study, we can evaluate the stroke impairment based on the involved activities without recording the sequence of performing the activities.

## 5. Conclusions

As sensors like accelerometers, gyroscopes, sEMGs, etc., gained widespread popularity and utilization in HAR, a comparative study to fully investigate the performance of these sensors was performed. These sensors were placed on the 12 key points of the upper/lower body limbs, and the optimal position for each kind of sensor under different groups in HAR was provided. Furthermore, in consideration of the practical scenario, a pre-trained HAR model on the healthy subjects was built and followed by the validation on the stroke survivors. The experimental results showed a great potential to use a single accelerometer attached to the extensor carpi ulnaris for the HAR applications.

## Figures and Tables

**Table 1:** The list of muscles attached with modules and the label abbreviation of each module.

Muscle	Sensor	Muscle	Sensor
Deltoid (anterior part)	US1	Tensor fasciae latae	LS1
Biceps brachii (short head)	US3	Rectus femoris	LS3
Brachioradialis	US5	Tibialis anterior	LS5
Deltoid (posterior part)	US2	Gluteus maximus	LS2
Triceps lateral head	US4	Biceps femoris longus	LS4
Extensor carpi ulnaris	US6	Gastrocnemius lateral	LS6

**Table 2:** Best classification accuracy (%) using sEMG, gyroscope, accelerometer, and their combination among 12 locations ${}^{1}$.

Classifier	Stroke Survivors				Healthy Subjects				All Subjects				Second Scenario			
	sEMG	GYRO	ACC	COMB	sEMG	GYRO	ACC	COMB	sEMG	GYRO	ACC	COMB	sEMG	GYRO	ACC	COMB
KNN	68.08 (3.17)	69.87 (5.66)	89.59 (3.64)	90.81 (3.18)	88.19 (3.91)	92.96 (2.09)	96.23 (2.41)	96.63 (2.3)	76.76 (3.99)	83.97 (2.96)	92.85 (1.91)	93.52 (1.79)	67.05 (4.55)	71.99 (5.88)	70.55 (4.79)	75.94 (4.96)
DT	64.59 (2.84)	67.95 (3.91)	90.64 (3.49)	90.57 (3.14)	87.8 (3.33)	90.97 (2.4)	95.83 (2.46)	95.34 (2.28)	78.56 (3.91)	82.31 (2.92)	91.12 (1.82)	94.34 (1.63)	69.86 (4.26)	70.85 (4.66)	72.53 (5.75)	72.9 (2.78)
AdaBoost	63.98 (5.17)	71.22 (4.03)	85.68 (2.37)	86.6 (3.35)	82.44 (3.95)	88.29 (2.64)	96.73 (2.11)	95.54 (2.44)	72.33 (3.31)	83.5 (2.78)	87.82 (2.42)	89.85 (2.0)	66.48 (3.77)	70.0 (5.65)	77.29 (3.86)	75.93 (4.18)
RF	67.99 (2.76)	72.64 (5.51)	92.14 (3.23)	92.45 (3.01)	86.41 (3.97)	92.56 (2.27)	96.33 (2.42)	96.53 (2.29)	77.6 (4.05)	84.31 (2.99)	93.73 (1.78)	94.43 (1.59)	69.18 (6.06)	74.29 (5.12)	76.41 (5.2)	74.37 (6.19)
LDA	70.33 (5.29)	74.8 (4.86)	91.29 (3.5)	92.94 (3.69)	87.5 (3.59)	92.96 (2.43)	96.23 (2.41)	96.53 (2.13)	78.13 (3.84)	84.62 (2.89)	92.98 (2.37)	94.73 (1.95)	70.27 (4.78)	71.42 (6.25)	73.73 (6.76)	74.7 (2.97)
ANN	67.74 (3.2)	74.38 (5.83)	91.82 (2.99)	93.42 (2.78)	88.29 (3.64)	92.96 (2.28)	96.23 (2.51)	96.73 (1.95)	79.94 (4.0)	85.5 (2.85)	94.48 (1.85)	95.95 (1.52)	72.35 (3.82)	75.12 (6.17)	74.78 (5.09)	77.75 (5.09)
GNB	68.03 (1.83)	71.69 (4.67)	90.38 (3.29)	91.46 (3.69)	85.32 (4.15)	89.58 (2.71)	94.94 (2.81)	96.33 (2.57)	75.78 (4.03)	82.22 (2.91)	87.62 (3.01)	91.23 (2.17)	68.42 (4.9)	72.0 (4.92)	67.02 (3.42)	72.83 (3.92)
SVM-Linear	71.16 (4.63)	72.23 (5.17)	91.18 (3.18)	92.58 (3.07)	86.9 (3.96)	93.45 (2.23)	96.13 (2.56)	97.82 (1.8)	78.74 (4.04)	85.13 (2.71)	94.51 (2.07)	95.85 (1.65)	70.52 (4.89)	73.99 (5.77)	72.23 (5.59)	77.7 (4.41)
SVM-RBF	73.77 (3.73)	75.88 (5.53)	94.05 (3.1)	94.22 (2.63)	90.87 (3.41)	94.54 (2.06)	96.43 (2.35)	96.53 (2.36)	84.09 (3.33)	87.95 (2.79)	95.84 (1.75)	96.56 (1.55)	76.34 (3.75)	76.82 (5.55)	77.89 (4.81)	82.47 (4.18)

^1^ The font in red color highlights the best classification accuracy among the involved classifiers. The value in parenthesis denotes the standard error of the accuracy.

**Table 3:** Pair-wise comparison of accelerometers in different positions for the group of all subjects ${}^{1}$.

Sensor	US6 (95.84)	US5 (94.92)	LS3 (90.72)	LS4 (89.19)	US3 (88.52)	US1 (87.58)	US4 (87.58)	US2 (86.17)	LS2 (85.44)	LS6 (84.85)	LS5 (83.08)	LS1 (82.45)
US6	-			**	***	***	**	***	**	**	***	***
US5	-	-		*	**	***	**	***	**	**	***	***
LS3	-	-	-					*	**	*	**	***
LS4	-	-	-	-					*	*	*	*
US3	-	-	-	-	-						*	*
US1	-	-	-	-	-	-						*
US4	-	-	-	-	-	-	-				*	
US2	-	-	-	-	-	-	-	-				
LS2	-	-	-	-	-	-	-	-	-			
LS6	-	-	-	-	-	-	-	-	-	-		
LS5	-	-	-	-	-	-	-	-	-	-	-	
LS1	-	-	-	-	-	-	-	-	-	-	-	-

^1^ The order of sensors was ranked based on their performances (from the best to the worst). The accuracy of each sensor was shown in the parenthesis under the sensor name on the first row. “-” denotes not filled, blank denotes there is no significant difference (*p* > 0.05) between related two items, * (0.05 > *p* > 0.01), ** (0.01 > *p* > 0.001), and *** (*p* < 0.001) denote there exists a significant difference between the related two items.

## Data Availability

Due to the privacy of the subjects, the data in this study are not publicly available.

## Abbreviations

The following abbreviations are used in this manuscript:

BS	Brunnstrom stage
HAR	human activity recognition
ADLs	activities of daily living
sEMG	surface electromyography
ACC	accelerometer
GYRO	gyroscope
COMB	combination
SMOTE	Synthetic Minority Oversampling Technique
mRMR	minimum-redundancy maximum-relevancy
LOSOCV	Leave-One-Subject-Out Cross-Validation
SVM	Support Vector Machine
RBF	Radial Basis Function
MI	mutual information
MID	Mutual Information Difference
W	walking
TB	tooth brushing
FW	face washing
D	drinking

## References

[1] Singh M., Pandey P.K., Bhasin A., Padma M.V., Mohanty S. (2020). Application of Stem Cells in Stroke: A Multifactorial Approach. Front. Neurosci..

[2] Kothari R., Sauerbeck L., Jauch E., Broderick J., Liu T.P. (1997). Patients’ awareness of stroke signs, symptoms, and risk factors. Stroke.

[3] Zambrana C., Idelsohn-Zielonka S., Claramunt-Molet M., Almenara-Masbernat M., Vargiu E. A hierarchical approach to recognize purposeful movements using inertial sensors: Preliminary experiments and results. Proceedings of the 11th EAI International Conference on Pervasive Computing Technologies for Healthcare.

[4] Nouri F., Lincoln N. (1987). An extended activities of daily living scale for stroke patients. Clin. Rehabil..

[5] Majumder S., Deen M.J. (2019). Smartphone sensors for health monitoring and diagnosis. Sensors.

[6] Majumder S., Deen M.J. (2020). A Robust Orientation Filter for Wearable Sensing Applications. IEEE Sens. J..

[7] Majumder S., Mondal T., Deen M.J. (2018). A simple, low-cost and efficient gait analyzer for wearable healthcare applications. IEEE Sens. J..

[8] Cook D., Feuz K.D., Krishnan N.C. (2013). Transfer Learning for Activity Recognition: A Survey. Knowl. Inf. Syst..

[9] Poppe R. (2010). A survey on vision-based human action recognition. Image Vis. Comput..

[10] Guiry J.J., Ven P.V.D., Nelson J., Warmerdam L., Riper H. (2014). Activity recognition with smartphone support. Med. Eng. Phys..

[11] Gu T., Chen S., Tao X., Lu J. (2010). An unsupervised approach to activity recognition and segmentation based on object-use fingerprints. Data Knowl. Eng..

[12] Cheng J., Chen X., Shen M. (2013). A Framework for Daily Activity Monitoring and Fall Detection Based on Surface Electromyography and Accelerometer Signals. IEEE J. Biomed. Health Inform..

[13] Majumder S., Mondal T., Deen M.J. (2017). Wearable sensors for remote health monitoring. Sensors.

[14] Xi X., Yang C., Shi J., Luo Z., Zhao Y.B. (2019). Surface Electromyography-Based Daily Activity Recognition Using Wavelet Coherence Coefficient and Support Vector Machine. Neural Process. Lett..

[15] Wu G., Xue S. (2008). Portable Preimpact Fall Detector With Inertial Sensors. IEEE Trans. Neural Syst. Rehabil. Eng..

[16] Gao L., Bourke A.K., Nelson J. (2014). Evaluation of accelerometer based multi-sensor versus single-sensor activity recognition systems. Med. Eng. Phys..

[17] Banos O., Toth M., Damas M., Pomares H., Rojas I. (2014). Dealing with the Effects of Sensor Displacement in Wearable Activity Recognition. Sensors.

[18] Lara O.D., Labrador M.A. (2013). A Survey on Human Activity Recognition using Wearable Sensors. IEEE Commun. Surv. Tutor..

[19] Lu J., Zheng X., Sheng M., Jin J., Yu S. (2020). Efficient human activity recognition using a single wearable sensor. IEEE Internet Things J..

[20] Tian Y., Wang X., Chen W., Liu Z., Li L. (2019). Adaptive multiple classifiers fusion for inertial sensor based human activity recognition. Clust. Comput..

[21] Mane S.M., Kambli R.A., Kazi F.S., Singh N.M. (2015). Hand Motion Recognition from Single Channel Surface EMG Using Wavelet & Artificial Neural Network. Procedia Comput. Sci..

[22] Cleland I., Kikhia B., Nugent C., Boytsov A., Hallberg J., Synnes K., McClean S., Finlay D. (2013). Optimal placement of accelerometers for the detection of everyday activities. Sensors.

[23] Olguın D.O., Pentland A.S. Human activity recognition: Accuracy across common locations for wearable sensors. Proceedings of the 2006 10th IEEE International Symposium on Wearable Computers.

[24] Maurer U., Smailagic A., Siewiorek D.P., Deisher M. Activity recognition and monitoring using multiple sensors on different body positions. Proceedings of the International Workshop on Wearable and Implantable Body Sensor Networks (BSN’06).

[25] Liparulo L., Zhang Z., Panella M., Gu X., Fang Q. (2017). A novel fuzzy approach for automatic Brunnstrom stage classification using surface electromyography. Med. Biol. Eng. Comput..

[26] Zhang Z., Fang Q., Gu X. (2014). Fuzzy inference system based automatic Brunnstrom stage classification for upper-extremity rehabilitation. Expert Syst. Appl..

[27] Xiong A., Guangmo L., Zhao X., Han J., Liu G. Feasibility of EMG-based ANN controller for a real-time virtual reality simulation. Proceedings of the IECON 2012—38th Annual Conference on IEEE Industrial Electronics Society.

[28] Majid J., Atena R.F., Katarzyna R., Zeljko Z. (2017). A Comprehensive Analysis on Wearable Acceleration Sensors in Human Activity Recognition. Sensors.

[29] Phinyomark A., Phukpattaranont P., Limsakul C. (2012). Feature reduction and selection for EMG signal classification. Expert Syst. Appl..

[30] Asim W., Nlandu K.E. (2018). Effect of threshold values on the combination of EMG time domain features: Surface versus intramuscular EMG. Biomed. Signal Process. Control.

[31] Sunil Babu M., Vijayalakshmi V. (2019). An Effective Approach for Sub-acute Ischemic Stroke Lesion Segmentation by Adopting Meta-Heuristics Feature Selection Technique Along with Hybrid Naive Bayes and Sample-Weighted Random Forest Classification. Sens. Imaging.

[32] Liu C., Ying W., Hao D., Yao R., Lin Y., Song Z., Zheng D. (2017). Effects of Force Load, Muscle Fatigue, and Magnetic Stimulation on Surface Electromyography during Side Arm Lateral Raise Task: A Preliminary Study with Healthy Subjects. BioMed Res. Int..

[33] Ramasso E., Placet V., Boubakar M.L. (2015). Unsupervised consensus clustering of acoustic emission time-series for robust damage sequence estimation in composites. IEEE Trans. Instrum. Meas..

[34] Klenk J., Becker C., Lieken F., Nicolai S., Maetzler W., Alt W., Zijlstra W., Hausdorff J.M., Lummel R.C.V., Chiari L. (2011). Comparison of acceleration signals of simulated and real-world backward falls. Med. Eng. Phys..

[35] Huang J., Hu P., Wu K., Zeng M. (2018). Optimal time-jerk trajectory planning for industrial robots. Mech. Mach. Theory.

[36] Chawla N.V., Bowyer K.W., Hall L.O., Kegelmeyer W.P. (2002). SMOTE: Synthetic Minority Over-sampling Technique. J. Artif. Intell. Res..

[37] Tan X., Su S., Huang Z., Guo X., Zuo Z. (2019). Wireless Sensor Networks Intrusion Detection Based on SMOTE and the Random Forest Algorithm. Sensors.

[38] Fernández A., Garcia S., Herrera F., Chawla N.V. (2018). SMOTE for learning from imbalanced data: Progress and challenges, marking the 15-year anniversary. J. Artif. Intell. Res..

[39] Nweke H.F., Teh Y.W., Mujtaba G., Alo U.R., Al-garadi M.A. (2019). Multi-sensor fusion based on multiple classifier systems for human activity identification. Hum. Centric Comput. Inf. Sci..

[40] Alharbi F., Ouarbya L., Ward J.A. Synthetic Sensor Data for Human Activity Recognition. Proceedings of the IEEE IJCNN.

[41] Cover T., Hart P. (1967). Nearest neighbor pattern classification. IEEE Trans. Inf. Theory.

[42] Castro D., Coral W., Rodriguez C., Cabra J., Colorado J. (2017). Wearable-based human activity recognition using an iot approach. J. Sens. Actuator Netw..

[43] Kumar R., Bayliff A., De D., Evans A., Das S.K., Makos M. Care-chair: Sedentary activities and behavior assessment with smart sensing on chair backrest. Proceedings of the 2016 IEEE International Conference on Smart Computing (SMARTCOMP).

[44] Xu L., Yang W., Cao Y., Li Q. Human activity recognition based on random forests. Proceedings of the 2017 13th International Conference on Natural Computation, Fuzzy Systems and Knowledge Discovery (ICNC-FSKD).

[45] Khan A.M., Lee Y.K., Lee S.Y., Kim T.S. Human activity recognition via an accelerometer-enabled-smartphone using kernel discriminant analysis. Proceedings of the 2010 5th International Conference on Future Information Technology.

[46] Suto J., Oniga S. (2018). Efficiency investigation of artificial neural networks in human activity recognition. J. Ambient Intell. Humaniz. Comput..

[47] Shen J., Fang H. (2020). Human Activity Recognition Using Gaussian Naïve Bayes Algorithm in Smart Home. J. Phys. Conf. Ser..

[48] Reyes-Ortiz J.L., Oneto L., Samà A., Parra X., Anguita D. (2016). Transition-aware human activity recognition using smartphones. Neurocomputing.

[49] Peng H., Long F., Ding C. (2005). Feature selection based on mutual information criteria of max-dependency, max-relevance, and min-redundancy. IEEE Trans. Pattern Anal. Mach. Intell..

[50] Ramírez-Gallego S., Lastra I., Martínez-Rego D., Bolón-Canedo V., Benítez J.M., Herrera F., Alonso-Betanzos A. (2017). Fast-mRMR: Fast Minimum Redundancy Maximum Relevance Algorithm for High-Dimensional Big Data. Int. J. Intell. Syst..

[51] Battiti R. (1994). Using mutual information for selecting features in supervised neural net learning. IEEE Trans. Neural Netw..

[52] Ding C., Peng H. (2005). Minimum redundancy feature selection from microarray gene expression data. J. Bioinform. Comput. Biol..

[53] Maaten L.V.D., Hinton G. (2008). Visualizing Data using t-SNE. J. Mach. Learn. Res..

[54] Potluri C., Kumar P., Anugolu M., Chiu S., Urfer A., Schoen M.P., Naidu D.S. sEMG based fuzzy control strategy with ANFIS path planning for prosthetic hand. Proceedings of the 2010 3rd IEEE RAS EMBS International Conference on Biomedical Robotics and Biomechatronics.

[55] Chen G., Wang W., Wang Z., Liu H., Li W. (2020). Two-dimensional discrete feature based spatial attention CapsNet For sEMG signal recognition. Appl. Intell..

[56] Jiang X., Xu K., Liu X., Dai C., Chen W. (2020). Neuromuscular Password-based User Authentication. IEEE Trans. Ind. Inform..

[57] Canning C.G., Ada L., Adams R., O’Dwyer N.J. (2004). Loss of strength contributes more to physical disability after stroke than loss of dexterity. Clin. Rehabil..

[58] Delph M.A., Fischer S.A., Gauthier P.W., Luna C.H.M., Clancy E.A., Fischer G.S. A soft robotic exomusculature glove with integrated sEMG sensing for hand rehabilitation. Proceedings of the 2013 IEEE 13th International Conference on Rehabilitation Robotics (ICORR).

[59] Zhang T., Fulk G.D., Tang W., Sazonov E.S. Using decision trees to measure activities in people with stroke. Proceedings of the 2013 35th Annual International Conference of the IEEE Engineering in Medicine and Biology Society (EMBC).

[60] Laudanski A., Brouwer B., Li Q. (2015). Activity classification in persons with stroke based on frequency features. Med. Eng. Phys..

[61] Lonini L., Gupta A., Kording K., Jayaraman A. Activity recognition in patients with lower limb impairments: Do we need training data from each patient?. Proceedings of the 2016 38th Annual International Conference of the IEEE Engineering in Medicine and Biology Society (EMBC).

